# RNA search engines empower the bacterial intranet

**DOI:** 10.1042/BST20160373

**Published:** 2017-07-14

**Authors:** Tom Dendooven, Ben F. Luisi

**Affiliations:** Department of Biochemistry, University of Cambridge, Tennis Court Road, Cambridge CB2 1GA, U.K.

**Keywords:** gene expression, riboregulation, RNA

## Abstract

RNA acts not only as an information bearer in the biogenesis of proteins from genes, but also as a regulator that participates in the control of gene expression. In bacteria, small RNA molecules (sRNAs) play controlling roles in numerous processes and help to orchestrate complex regulatory networks. Such processes include cell growth and development, response to stress and metabolic change, transcription termination, cell-to-cell communication, and the launching of programmes for host invasion. All these processes require recognition of target messenger RNAs by the sRNAs. This review summarizes recent results that have provided insights into how bacterial sRNAs are recruited into effector ribonucleoprotein complexes that can seek out and act upon target transcripts. The results hint at how sRNAs and their protein partners act as pattern-matching search engines that efficaciously regulate gene expression, by performing with specificity and speed while avoiding off-target effects. The requirements for efficient searches of RNA patterns appear to be common to all domains of life.

## Introduction

Like its eukaryotic and archaeal counterparts, bacterial gene expression is regulated by a multitude of mechanisms. At the level of mRNA synthesis, regulation is mediated by transcription factors and their partners, whose actions are often organized into co-operative networks. These networks generate co-ordinated patterns of expression and underpin the orchestrated responses to signals and changing metabolic conditions or help to launch multistep programmes, such as the generation of enormous multicomponent assemblies (e.g. the bacterial flagellar machinery) [1].

Although transcriptional networks have regulatory power and versatility, additional processes must be involved in achieving stable control. One indication that there is more to the control of gene expression than the regulation of transcription alone is the often-noted discrepancy in abundances of mRNAs and cellular proteins [2–6]. The additional regulatory processes, which have been experimentally confirmed to occur post-transcription, represent another critical stage of genetic control. For instance, processes that affect translation initiation contribute to post-transcriptional control [7,8]. Another key control parameter post-transcription is mRNA lifetime [9], which can be modulated in numerous ways, including ribosomal masking [10], prevalence of endonucleolytic cleavage sites, cellular levels, and activity of ribonucleases [11–13], addenda at the termini of mRNAs and other post-transcriptional modifications [14–16], regulatory effectors that control ribonuclease-binding affinities, cellular location, and interactions with regulatory RNAs [17–19].

**Figure 1.**
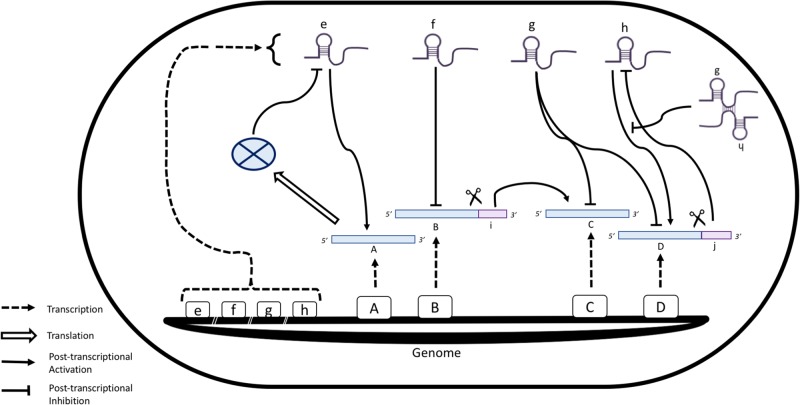
Schematic representation of known bacterial sRNA-mediated control mechanisms in idealized sRNA-mediated post-transcriptional regulatory networks. sRNAs are represented by hairpin structures; mRNAs and their coding genes are depicted by blue bars and black boxes, respectively. sRNAs can either enhance (e) or inhibit (f) translation, depending on the context. The mRNA of gene A encodes a representative ribonuclease (blue oval with cross) that degrades sRNA e, which, in turn, targets mRNA A, together forming a negative feedback loop over a transcriptional and translational level. A single mRNA (D) can be targeted by several sRNAs (g and h), but one sRNA (g) can equally target several mRNAs (C and D). Precursor sRNAs can be generated from 3′ mRNA tails by endonuclease cleavage (i and j) [40]. Moreover, ‘Sponge’ interactions between g and h can inhibit the activity of h by sequestration, further adding to the complexity of the sRNA interactome. The schematic is not exhaustive, but intends to convey the potential richness of the RNA-mediated control networks.

Many elegant experimental studies over the last decade have revealed that small regulatory RNAs (sRNAs) are central factors for post-transcriptional control in diverse bacterial and archaeal species, in which they influence RNA stability, processing, and translation [20]. The activities of sRNA molecules in post-transcriptional regulation are often found to be organized into networks that rival the complexity and deep connectivity of their counterparts in transcription control (see Figure 1) [21]. The RNA-mediated regulatory networks participate in nuanced responses to multiple inputs, including developmental cues or signals arising from stress or changes in metabolic state. sRNAs have been shown to also play important roles in the programmes of host invasion by pathogens, including the cell-to-cell quorum-sensing communications that co-ordinate infections by *Vibrio cholera*, the cholera-causing bacterium [20,22]. Even though sRNAs are ubiquitous in bacteria and archaea [20], their mechanism and function have been particularly well studied in species such as *Escherichia coli* and the closely related *Salmonella*. These enteric gamma-proteobacteria have been shown to encode hundreds of sRNAs that bear a region of partial or complete sequence complementarity to target transcripts, known as the ‘seed region’ [20]. When mRNAs are bound by a partner sRNA, their translation efficiency is altered, or they are directed to an irreversible fate of rapid turnover. Whether an sRNA boosts or suppresses translation often depends on whether target interaction exposes or sequesters the translation initiation element [23,24]. Many parallels can be drawn between bacterial sRNAs and their metazoan counterparts: the miRNAs that target transcripts to direct post-transcriptional repression, either via translation inhibition or destabilization of the mRNA [25]. The components of the bacterial and eukaryotic RNA-based control systems do not appear to share evolutionary ancestry, and their convergent properties highlight the fitness benefit of RNA-mediated regulation throughout different domains of life.

RNA-mediated control (‘riboregulation’) bears loose analogy to information retrieval by an internet search engine, whereby a user-defined query text will return web page hits ranked based on probabilistic matches to that text. sRNAs, much like a search query, can be used to scan the mRNA population for matches through complementary base-pairing. The quality of the resulting matches is based on sequence similarity, the length and number of sRNA complementary regions, as well as structural features and cellular abundances of both sRNA and mRNA species. One of the puzzles concerning RNA-mediated regulation is how specificity is achieved, and off-target effects are avoided, given that the seed region often pairs to a target with imperfect base-pairing complementarity over, sometimes, very short segments that are not thermodynamically stable in isolation, and considering the astronomical numbers of potential interaction sites with entirely wrong RNA partners. Part of the solution rests in the properties of the ribonucleoprotein effector complexes involving sRNAs. In bacteria, RNA chaperones, ribonucleases, and other helpers facilitate the search to match sRNA to target and deliver the optimal functional output. These helper proteins accelerate the pairing between sRNA and mRNA and provide an additional level of specificity [20,26,27], much like the behaviour seen for eukaryotic RNA-targeting ribonucleoprotein complexes [28,29].

Here, we will describe recent advances in understanding how sRNAs are recognized and paired with mRNAs in bacteria, *in vivo*. We will explore a representative RNA degradation machinery that can be programmed by RNA regulators and describe the process of sRNA-mediated decay. The RNA chaperones that facilitate the process of RNA action will be presented.

## A regulatory hub of RNA metabolism

In *E. coli* and related bacterial species, an important regulatory hub of post-transcriptional gene regulation is the RNA degradosome, a multienzyme machine that functions in RNA processing and turnover as well as in sRNA-mediated target silencing. In many ways, it is functionally analogous to the miRNA/siRNA RISC complex (the RNA-induced silencing complex) of eukaryotes [28,29].

The composition of the bacterial RNA degradosome varies in the course of evolution and, even within one species, changes with growth phase and environmental conditions [30]. The canonical ‘core’ unit of the extensively studied *E. coli* RNA degradosome comprises the following enzymes: the hydrolytic endonuclease RNase E, which is the main component and scaffold; the phosphorolytic exoribonuclease polynucleotide phosphorylase (PNPase); an ATP-dependent helicase RhlB, and enolase, a glycolytic enzyme [31–33]. These degradosome components co-operate to act as an integrated molecular machine [34]. The functional importance of the degradosome is suggested not only by its widespread occurrence in diverse bacterial species, but also by the finding that analogous machinery has arisen through convergent evolution in the Gram-positive bacterial lineages (as represented by the ribonuclease-based assemblies of *Bacillus subtilis* and the pathogenic species *Staphylococcus aureus* [35–37]).

For most *E. coli* mRNAs, turnover rapidly follows after cleavage by the main degradosome component, RNase E. The enzymatic activity of RNase E can be substantially boosted if the 5′-end of the substrate is mono-phosphorylated. Nascent transcripts are produced with a 5′-triphosphate group, but this can be converted into a monophosphate by an RNA pyrophosphohydrolase (such as RppH; [38]). Following RNase E cleavage of RNA, PNPase then further degrades the product in the 3′ → 5′ direction, and remaining fragments are reduced to single nucleotides by oligoribonuclease [33]. The ATP-dependent RNA helicase partner of RNase E, RhlB, is a DEAD-box family member that unwinds double-stranded RNA species or remodels protein–RNA interactions to facilitate degradation [32,39]. Thus far, a functional role for enolase in the degradosome has not been found, although some experiments point towards the enzyme being an energy sensor controlling the stability of mRNAs that encode proteins involved in energy pathways [32,41,42] and carbon utilization [43].

**Figure 2.**
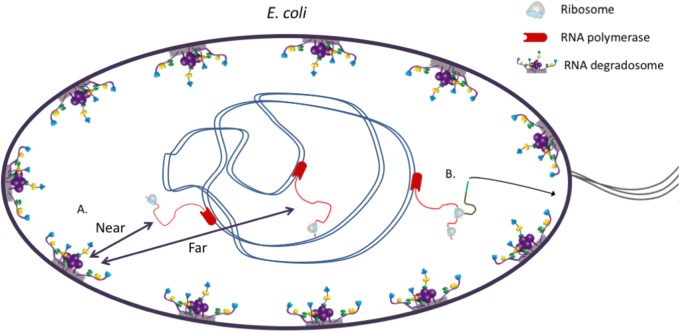
The membrane association of the RNA degradosome potentially adds an extra layer of post-transcriptional control to gene regulation by introducing a spatially encoded intrinsic time delay [49]. The intrinsic time delay can also be encoded by individual diffusion rates. A: mRNAs (red lines) that are transcribed by RNA polymerase (red shapes) at the periphery of the genome (blue) are closer to the RNA degradosomes (purple), and therefore speculated to be less stable than transcripts that are expressed in the centre of the bacterial genome (black double arrows) [17]. Ribosomes are drawn in light blue. B: Alternatively, mRNAs encoding inner membrane proteins (dark green) are directed to the membrane co-translationally by a signal peptide (light green), which reduces their stability [48]. The components are not drawn to scale.

The C-terminal portion of RNase E serves as a recruiting scaffold for the other components of the degradosome. The catalytic, globular N-terminal domain forms a homotetramer that is organized as a dimer of dimers [44]. Adjacent to the N-terminal catalytic domain in the sequence is an amphipathic α-helix that tethers the RNA degradosome to the bacterial cell membrane in *E. coli* [19,45] and is expected to affect the way that the four natively unstructured C-terminal regions would extend outwards the tetrameric catalytic centre (see Figure 2). Strikingly, the functionally analogous (but not homologous) enzyme of *S. aureus*, RNase Y, is also membrane-associated [46]. Although the association of RNase E with the cytoplasmic membrane is required for optimal cell growth in *E. coli*, the membrane localization of RNA degradosomes is not ubiquitous in bacteria. For instance, in *Caulobacter crescentus*, the degradosome assembles into foci close to the bacterial chromosome [47].

It seems likely that RNA stability could, in part, be controlled by its cellular location and proximity to the RNA decay machinery. Thus, the subcellular localization of the RNA degradation machinery in *E. coli*, *S. aureus,* and other bacterial species could, in principle, add an additional layer of organization to RNA-mediated regulation. Insights into the spatial organization of cellular RNA and its potential relationship to post-transcriptional control have recently been provided using super-resolution microscopy [48]. Interestingly, mRNAs that encode proteins from the inner membrane localize at the membrane in *E. coli*. Moreover, these mRNAs are subject to higher degradation rates, which are abolished when the RNA degradosome is dissociated from the membrane via point mutations in the membrane attachment amphipathic helix. As for the features that might induce membrane enrichment, it was shown that localization is conferred co-translationally by signal peptides that direct the mRNA species to the membrane. Artificially inducing membrane localization of several mRNAs by incorporation of sequences, encoding such signal peptides, reduced their stability significantly [48]. Finally, both activating (e.g. GlmZ) and inhibitory sRNAs (OxyS, RyhB, and SgrS) were shown to preferentially localize in the nucleoid and cytoplasm in *E. coli*, whereas a mRNA control (*gfp*) is rarely present in the nucleoid, most likely because of its larger size compared with sRNAs [50]. These findings further hint towards a spatial level of post-transcriptional control in the cell. It is interesting to note that sRNAs do not seem to have preferential membrane localization, even though the membrane-localized RNA degradosome is the main degrading machinery for sRNA-tagged mRNAs in *E. coli*. Conceivably, this could be due to rapid degradation of sRNA–mRNA pairs at the membrane. In light of the membrane association of the degradosome, it is possible that mRNAs that are transcribed at the periphery of the *E. coli* genome (and therefore closer to the bacterial cell membrane) may be more prone to degradation than RNA species in which the corresponding genes are localized more centrally (see Figure 2) [17].

One interesting finding related to membrane association of the *E. coli* degradosome is that the assembly is highly mobile on the membrane surface, but forms transient punctuate loci that are likely to be centres of RNA turnover [51]. These loci share remarkable similarities and potential functional analogy with the eukaryotic ribonucleoprotein (RNP) granules formed by RNA-binding and -processing enzymes. The eukaryotic RNP granules are microscopic structures resembling phase-separated droplets and are proposed to act as ‘nano-organelles’ that are partitioned from the cytoplasm without the requirement for a lipid membrane [51]. The liquid–liquid phase separation is postulated to be mediated by disordered regions of RNA-binding proteins that can form new interactions within such droplets. The granules compartmentalize enzymes and RNA-binding proteins, and also influence their specificities for nucleic acids. In the context of the degradosome, extensive unstructured regions in the C-terminal tail of RNase E could promote loci formation through self-interaction or distributed contacts with RNA. The transient degradosome loci on the cytoplasmic membrane could yield highly co-operative behaviour of enzyme activities on a bound substrate. It is interesting that eukaryotic helicases, such as mammalian DDX3 and yeast Ded1 DEAD-box, are recruited into cytoplasmic bodies [52], where they likely play roles in restructuring RNA or remodelling RNA–protein complexes. Perhaps, the DEAD-box helicase of the degradosome, RhlB, plays a similar role in the context of the transiently formed membrane loci.

## Target recognition and presentation by small RNAs

Accumulating evidence supports a model in which sRNAs are the main regulatory factors for post-transcriptional control in diverse bacterial species, where they influence RNA stability, processing, and translation [20]. sRNAs are typically 50–200 nucleotides in length and differ greatly in predicted structure, but share in common the capacity to form base-pairing interactions with their target transcripts using only a short ‘seed’ region. Imperfections in the match of seed and target are permitted and may even be favoured. sRNAs typically interact with the 5′-end of a transcript and are often degraded together with their target. The identification of sRNAs [53] and mapping their targets [54,55], widely referred to as the ‘sRNA interactome’, have rapidly advanced with the continuous development of RNA sequencing methods.

**Figure 3.**
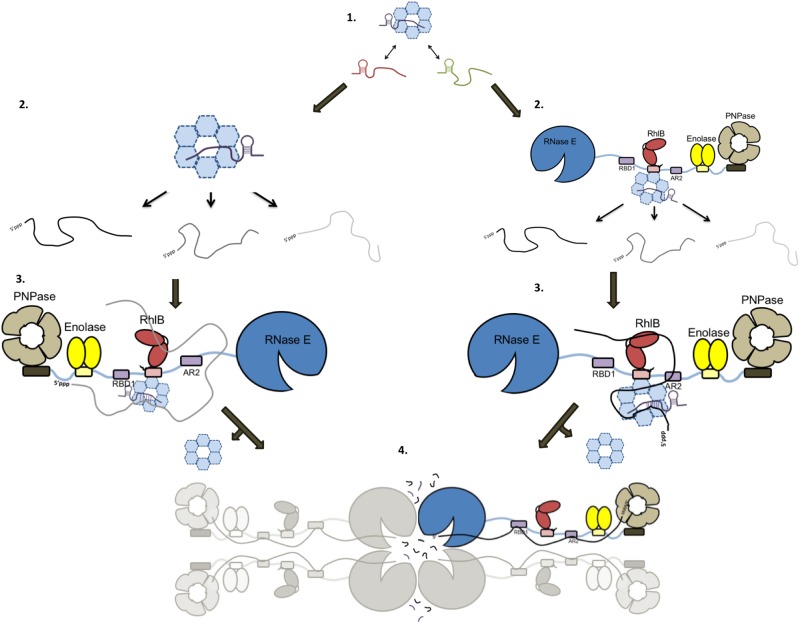
Two speculative sRNA-mediated mRNA decay processes by the RNA degradosome. Hfq transiently binds different sRNA species, maintaining a dynamic equilibrium between unbound and Hfq-associated sRNAs (1). (Left) The Hfq:sRNA complex screens the cellular mRNA library for targets with sufficient sequence complementarity to the sRNA seed region (2). When a target is captured, the sRNA:mRNA:Hfq complex binds the RNA degradosome (depicted as a protomer) (3) after which the Hfq is rapidly displaced by RNase E, and the sRNA:mRNA duplex binds the RBD and AR2 RNA-binding sites, followed by degradation by the RNase E catalytic domain (4) [55,63]. (Right) In an alternative decay model, the sRNA:Hfq duo interacts with RNase E first, before interrogating the cellular mRNA in association with the RNA degradosome (2). Once a suitable mRNA target is found, the sRNA:mRNA duplex is handed over to RNase E by Hfq, after which the sRNA:mRNA pair binds the RNA-binding sites on the RNase scaffold domain, followed by degradation by the RNase E catalytic domain (3 and 4). Hfq is presented as a dark blue hexamer. RNase E is depicted in light blue, RhlB in red, Enolase dimers in yellow, and PNPase trimers in grey. RNA-binding sites are displayed as purple boxes on the RNase E scaffold domain. In the section ‘New roles for old players: sRNA chaperones and modulators’, the RNA degradosome is depicted in its native tetrameric form to highlight how the catalytic domains come together to form a central, degrading core complex (figure adapted from [90]).

Many sRNAs require an association with modulators and chaperones in order to properly control post-transcriptional gene expression. The strategy of controlling the search for targets by presenting short, complementary seed regions in a ribonucleoprotein complex is found in numerous RNA-mediated regulatory pathways of eukaryotes, bacteria and archae, for example in RNA interference and CRISPR-CAS systems [56]. The most common of these modulators in bacteria is the ring-like, hexameric RNA chaperone Hfq, a member of the extensive Lsm/Sm protein family [57,58]. In *E. coli* and *Salmonella enterica*, Hfq and Hfq-dependent sRNAs regulate more than 20% of all gene expression [59,60]. Hfq promotes sRNA–mRNA duplex formation [61,62], protects sRNA from degradation by ribonucleases [58], and recruits RNase E to mediate the decay of target mRNAs (see Figure 3) [63,64].

Recently, the targets of sRNAs mediated through Hfq have been elucidated on a transcriptome-wide scale [54,59]. Exploiting the newly developed methodology of RIL-seq (RNA interaction by ligation and sequencing), interactions have been captured between Hfq-­associated sRNAs and their targets *in vivo*, revealing an extensive sRNA-target network and tremendously expanding the current atlas of the *E. coli* sRNA interactome [54]. The network is highly dynamic, and extensive rewiring occurs with changing cellular conditions, such as iron limitation and different growth phases. Moreover, the actions of sRNAs can be balanced through decoys (or ‘sponges’), identified in the RIL-seq analysis and other studies [65,66], that sequester the sRNAs through base-pairing interactions (see Figure 1). A recent CLIP-seq (cross-linking and immunoprecipitation and sequencing) study revealed 3′-Rho-independent terminators in both sRNAs and mRNAs as a global recognition site for Hfq in *Salmonella* [67,68]. The results show that Hfq targets 5′ sRNA-binding sites in mRNAs and regions close to seed sequences in sRNAs. These data support a model whereby Hfq is transiently sandwiched between the mRNA and sRNA of cognate RNA pairs and helps to mediate RNA duplex formation between the two RNAs [69–71].

A sequencing-based study has elucidated the role of RNase E in the maturation of Hfq-dependent sRNAs in *S. enterica* Typhimurium [40]. By aligning thousands of cleavage sites, a minimal RNase E consensus sequence was identified as RN_WUU (with N as any nucleotide, R as G/A and W as A/U), with a significant preference for uridine at the +2 position [40]. RNase E cleavage sites were enriched in non-coding RNA precursors and in 3′-UTRs (untranslated region) of mRNA transcripts (see Figure 1). In fact, RNase E cleavage at the 3′-UTR sites can generate active sRNAs. Additionally, RNase E-mediated maturation was found to be crucial for target regulation by Hfq-dependent RNAs, as demonstrated for the action of the ArcZ sRNA towards its target transcript (*tpx*).

Further insights into the physical proximity of protein and RNA molecules involved in sRNA regulation were achieved with a variant on RNA sequencing, the CLASH methodology (UV-cross-linking, ligation, and sequencing of hybrids) [55,72,73]. By analyzing the sequence regions of hundreds of cognate RNase E-binding sites on sRNA species, the Hfq maximal binding regions on these sRNAs were found on average to be 5 nt to the 5′-side of the RNase E maximal binding regions, with significant overlap. In addition, oligo(A) tails, which are tags often added following RNase E cleavage, were mostly present 13 nt to the 3′-side of the RNase E maximal binding site. Furthermore, seed sequences have been identified within several sRNAs and the complementary motifs in their target RNAs, making it clear that not only can distinct RNA species be targeted by the same seed region, but also multiple sRNAs can target the same RNA (see Figure 1) [27,55]. The seed sequences were again strongly overlapping with Hfq-binding sites. These data support an interaction/displacement model whereby RNase E binds closely to Hfq interaction sites on sRNAs, thereby displacing Hfq from the sRNA-target RNA pair (see Figure 3), followed by cleavage 13 nt downstream and an elongation of the cleaved transcript with an oligo(A) tail. This model is consistent with the RNase E cleavage 6 nt downstream from the MicC-*ompD* sRNA–mRNA duplex [55,63].

A novel sRNA-mediated mechanism for gene activation on a transcriptional level has also been revealed through deep-sequencing studies [74]. In some cases, Hfq-associated sRNA molecules can bind the 5′-UTR of elongating transcripts to prevent Rho-dependent premature termination. The transcription of *rpoS*, which encodes the stress factor σ^S^, is strongly increased via this sRNA-mediated antitermination mechanism upon entry into the stationary growth phase, conceivably to help accommodate the associated metabolic changes within the cell. The sequencing results suggest sRNA-mediated antitermination as a widespread transcriptional control mechanism of Rho-dependent termination, adding another mode to the repertoire of sRNA-mediated riboregulation.

RNA sequencing-based techniques have led to a tremendous increase in our understanding of the sRNA interactome both on a global level, with the discovery of numerous new sRNAs and their targets, and at ‘high-resolution’ levels, with the elucidation of the exact mechanisms and recognition patterns that drive the sRNA-mediated decay.

## New roles for old players: sRNA chaperones and modulators

The role of Hfq as a global sRNA chaperone in bacteria has been well established, but given its estimated cellular numbers and anticipated workload, it is not expected to be the sole protein fulfilling a chaperone function. Many sRNAs are associated with members of the CsrA/RsmA protein family. CsrA, a translational repressor that targets hundreds of mRNAs, is sequestered by the sRNAs, McaS, and CsrB/C [75,76]. CsrA binds GGA motifs in loops of stem-loop structures in mRNA 5′-UTRs as well as in some sRNAs [77]. The CsrB sRNA, for example, contains multiple hairpin structures, enriched in GGA sequences. CLIP-seq results, however, suggest that a longer AUGGA motif in apical loops of hairpin structures is the global recognition pattern for CsrA [68]. Finally, the CLIP-seq experiments and functional assays have shown that CsrA controls mRNAs coding for *Salmonella* virulence factors [68].

Notably, many sRNAs in *E. coli* and *S. enterica* lack Hfq or CsrA recognition motifs [67,68], suggesting that there may be other chaperones involved in riboregulation. Potentially filling this chaperone gap is the RNA-binding protein ProQ, which was recently discovered to be an important sRNA-binding protein involved in post-transcriptional gene expression [78]. The sRNAs bound by ProQ show little overlap with the Hfq and CsrA-associated sRNA pools [77]. ProQ was originally identified as an osmoregulatory protein that controls the expression of ProP, a proline channel, but is predicted to be as abundant as the highly expressed Hfq and CsrA RNA chaperones [78,79]. Upon ProQ deletion, the abundance of nearly a thousand transcripts was affected, suggesting the presence of a formerly unknown ProQ-based regulon for post-transcriptional gene expression [77]. Grad-seq analyses (gradient profiling by sequencing) revealed close to 100 ProQ-associated sRNAs in *S. enterica*, most of which are Hfq-independent, suggesting that ProQ associates with a new class of highly structured sRNAs [78]. The structure of ProQ has recently been shown to adopt an elongated rod shape, and it has been proposed that elongated RNA targets of ProQ can bind along the length of this rod structure [80]. Among the identified ProQ-enriched sRNAs were an attenuator (SraF; [81]), an sRNA sponge (STnc2180; [65]), and several type I antitoxins (Sib, Rdl, and IstR; [82,83]). In a recent study by Smirnov et al. [84] on the ProQ-dependent sRNA RaiZ, the regulatory role for ProQ was found not only to stabilize RaiZ, but also to actively prevent the 30S ribosome from loading on the RaiZ target, the *hupA* mRNA. As such, ProQ forms a ternary complex with the RaiZ–*hupA* duplex, whereas in certain proposed decay models Hfq is believed to only bind single-stranded RNA species (see Figure 3). ProQ homologues may play roles in riboregulation in diverse bacterial species. For instance, a ProQ/FinO domain containing regulator in the human pathogen *Legionella pneumophila* was shown to associate with a *trans*-acting sRNA, RocR, to repress the expression of the DNA uptake machinery [85].

Surprisingly, the exoribonuclease PNPase may play a cryptic chaperone role, even though the main role of PNPase is to degrade RNAs in *E. coli* and *S. enterica* [86–88]. De Lay and Gottesman [89] found that PNPase is required to stabilize some sRNA species *in vivo*, and, based on functional experiments, have proposed a protective role for PNPase for some sRNA species. Andrade et al. [88] further reported that some sRNAs are stabilized by PNPase in a growth phase-dependent manner. PNPase was shown to sequester sRNAs from other ribonucleases without degrading them, binding at least 11 of 24 known Hfq-associated sRNAs [86]. Finally, PNPase and Hfq could form a ternary complex with sRNAs *in vivo*, in which PNPase is unable to degrade sRNAs. As such, sRNAs could gain cumulative protection from both Hfq and PNPase in this ternary complex, being fully protected only when both modulators are present. Both the S1 RNA-binding domain and the active site of PNPase play a role in binding sRNAs, although more determinants are likely to be involved. As to how PNPase would interact with sRNAs in the ternary complex, one possibility is that the enzyme starts degrading the 3′-end of a Hfq-associated sRNA, but is stalled at this end, forming a shielding Hfq–PNPase–sRNA complex [86,91].

## Summary and perspective

The interactions between sRNAs, ribosomes, ribonucleases, RNA chaperones, and target mRNAs affect the kinetics and efficiency of riboregulation *in vivo*. There has been much progress towards explaining how the key ribonuclease of riboregulation, RNase E, recruits and acts upon sRNA:mRNA pairs. Numerous molecular genetics studies have revealed that RNase E and the RNA chaperone Hfq physically associate *in vivo* and work in conjunction in numerous cases of sRNA-mediated target silencing [42]. Moreover, RNA sequencing following cross-linking revealed sRNA:mRNA pairs associated with RNase E [55]. The choreography of events in this riboregulation process is still to be established. Does RNase E within the degradosome capture the pre-formed Hfq/sRNA complex and then present this to interrogate mRNA, perhaps through an active threading process? Or does a Hfq:sRNA complex engage a target mRNA prior to binding to the degradosome? In either scenario, once an mRNA partner is found, the duplex RNA can be displaced from Hfq for handover to the catalytic domain of RNase E. Recent evidence indicates that the duplexes formed between an sRNA and a target transcript can be displaced by the natively unstructured C-terminal tail of the RNA chaperone Hfq chaperone [62], and this would facilitate the envisaged duplex handover (see Figure 3).

The expanding studies into bacterial riboregulation are rapidly providing a detailed atlas of the dynamic pairings that occur *in vivo* and the machinery involved. These underpin the accurate and fast search modes that enable bacteria to make rapid and robust responses to environmental and developmental stimuli. As these data become increasingly complemented with *in vivo* kinetic studies [92], a comprehensive analysis of the steps involved in the complex and high fidelity mechanisms of the bacterial riboregulation will unfold. As our knowledge of the bacterial RNA intranet grows, it will become more apparent how such systems originate and how they can accommodate change in the course of evolution [93].

## Abbreviations

CLIP-seq: cross-linking and immunoprecipitation and sequencing
PNPase: phosphorolytic exoribonuclease polynucleotide phosphorylase
RNP: ribonucleoprotein
sRNAs: small RNA molecules
UTRs: untranslated region
